# Cultural and Technological Barriers to Telehealth Adoption for Type 2 Diabetes Management Among Asian American Patients: Qualitative Case Study

**DOI:** 10.2196/75689

**Published:** 2026-02-03

**Authors:** Devi Gurung States

**Affiliations:** 1 Himalayan Family Healthcare Project (HFHCP), 4145 Manchester Ave, St. Louis, MO, United States, 1 (314) 899-7590; 2 Peabody College of Education and Human Development, Vanderbilt University, Nashville, TN, United States

**Keywords:** Asian American, type 2 diabetes, telehealth, digital literacy, cultural barriers, health disparities

## Abstract

**Background:**

In the past decade, telehealth has transformed health care delivery by allowing patients more rapid and convenient access to necessary care without the cost and logistical challenges of traveling to a health care facility. Telehealth services can benefit patients with type 2 diabetes mellitus (T2DM) amid a growing epidemic of T2DM in the United States that affects people of all ages and races. In 2020, 33 million people were diagnosed with this chronic disease, with the number expected to rise by 50% by 2040. Telehealth facilitates regular contact between patients and their providers, especially when there are geographic barriers and time constraints prohibiting physical interaction, at little or no added cost to the patient and at their convenience.

**Objective:**

This study examines cultural and technological barriers affecting telehealth adoption among Asian American people with T2DM.

**Methods:**

A qualitative case study approach was employed, utilizing semistructured interviews with 30 Asian American individuals in Missouri. Thematic analysis was used to identify key barriers.

**Results:**

Four major barriers emerged: (1) language and cultural barriers—limited availability of translated materials and interpreters; (2) limited digital literacy and access—older adults and individuals with low technological exposure struggled with telehealth platforms; (3) limited provider recommendations—health care providers did not actively endorse telehealth, reducing patient awareness of telehealth as an option; and (4) technology access and infrastructure disparities—low-income participants faced challenges with the costs of and access to broadband and telehealth-compatible devices.

**Conclusions:**

Addressing cultural and technological barriers is crucial to increasing telehealth adoption among Asian American people with T2DM. Culturally tailored interventions, provider engagement, and digital literacy programs should be prioritized. Policy efforts must focus on expanding broadband access and providing multilingual telehealth resources.

## Introduction

Telehealth refers to a tool, process, or system that can provide patients with a simpler appointment scheduling process, remote access to clinical services that lessen the need for travel, and increased interaction with health care providers to improve medical outcomes while reducing health care costs [1,2]. In the past decade, telehealth has transformed health care delivery by allowing patients more rapid and convenient access to necessary care without the cost and logistical challenges of traveling to a health care facility [3]. Telehealth services can benefit patients with type 2 diabetes mellitus (T2DM) amidst a growing epidemic of T2DM in the United States that affects people of all ages and races [4]. In 2020, 33 million people were diagnosed with this chronic disease, with the number expected to rise by 50% by 2040 [4].

Telehealth facilitates regular contact between patients and their providers, especially when geographic barriers and time constraints prohibit physical interaction, at little or no added cost to the patient and at their convenience [5]. Telehealth services have the potential to optimize T2DM management by empowering patients to engage in self-care to slow disease progression, prevent complications, and lessen the health care burden [4,6]. Self-management among patients with T2DM includes proper nutrition, adequate physical activity, regular blood glucose monitoring, medication compliance, disease knowledge, lifestyle modifications, and self-efficacy [7]. To ensure effective self-management among patients at high risk or with a diagnosis of T2DM, telehealth can facilitate digital health coaching for long-term management or prevention of T2DM across population subgroups [8]. Asian American people include multiple subgroups (eg, Chinese, Indian, Vietnamese, Filipino, Korean, and Nepali). Cultural differences among these groups are substantial, especially among first-generation individuals.

According to the US Department of Health and Human Services [9], Asian American people are 40% more likely to be diagnosed with diabetes than non-Hispanic White individuals. Despite this higher prevalence, telehealth utilization among Asian American people remains low, as this population is disproportionately underserved [6,10]. Several factors contribute to this disparity, including cultural norms, digital literacy, provider engagement, and technological access [11]. Many Asian American communities face language barriers, limiting their ability to navigate telehealth platforms effectively [12,13], and older adults may struggle with technological proficiency, creating challenges in virtual health care engagement.

Given the disproportionate burden of T2DM among Asian American people and their persistently low telehealth usage, this study sought to answer the following questions: what cultural and technological barriers limit adoption? and what strategies might address them? These questions are urgent given the stakes, including T2DM complications, loss of care access, and widening racial health disparities. By centering user experience and structural constraints, the study identifies critical leverage points for more equitable digital health policy and practice.

Understanding these barriers is critical for designing equitable telehealth interventions that improve access to diabetes care. Therefore, this study explored the cultural and technological challenges hindering telehealth adoption among Asian American people with T2DM, providing insights for health care providers and policymakers. The guiding theoretical framework was the unified theory of acceptance and use of technology (UTAUT), an integrated approach used to predict and understand how and why individuals or groups accept or reject various technologies [11]. In the context of this inductive study, UTAUT provided a foundation for examining telehealth adoption among Asian American people with T2DM and was used to scaffold the study. UTAUT was used to identify several characteristics that influence the adoption of health technologies, which included social influence, performance expectancy, effort expectancy, facilitating conditions, privacy risk, and the threat of the disease from which the patient is ailing. By investigating factors affecting telehealth use within this population, specifically for the management of T2DM, a greater understanding of barriers to adoption of telehealth services and associated effects was achieved. Depending on the applicability of this study’s findings to specific Asian American communities, this knowledge can be applied to support the development of more effective methods of tailoring telehealth services to meet the unique needs of Asian American people, as well as those of other underserved populations. This study provided insights regarding ways to incorporate culturally relevant telehealth approaches to T2DM management into mainstream health care practice.

In addition to the UTAUT framework, this study was informed by a critical health equity lens that emphasizes how structural inequities—such as limited English proficiency, systemic underinvestment in minority-serving institutions, and digital exclusion—shape health care access. This critical perspective extends UTAUT by interrogating not only users’ perceptions of telehealth but also the sociotechnical structures that enable or constrain its use among marginalized populations. By situating Asian American T2DM management within broader systems of racialized health care access, this study provides an intersectional understanding of telehealth adoption.

## Methods

### Study Design and Researcher Perspective

A qualitative case study approach was used to explore telehealth adoption barriers among Asian American people with T2DM in Missouri. The purpose of the study was to examine Asian American people’s perspectives to determine the factors that influence their adoption of telehealth for T2DM management. Regarding positionality, my interest in this demographic is that I am an Asian American. Subjective perspectives, rather than objective facts, were elicited from participants; therefore, a qualitative methodology was appropriate, as it enabled in-depth examination [14].

The study focused on Asian American people with T2DM in Missouri due to the group’s elevated diabetes risk and underrepresentation in telehealth research. Missouri, as a Midwestern state with rising Asian American populations [15], offered a novel geographic and demographic context. A qualitative case study design was used to capture the complexity of individual, cultural, and technological factors shaping telehealth use, particularly within a population that often faces intersecting language and access barriers. This approach aligns with case study methodology’s strengths in revealing nuanced insights within specific, bounded systems.

The study population included Asian American people with T2DM via convenience sampling [16]. Convenience sampling may overrepresent individuals with similar socioeconomic status or community ties and introduce selection bias. Inclusion criteria specified that participants be Asian American, 21 years or older, and diagnosed with T2DM. Those with type 1 diabetes mellitus and gestational diabetes were excluded, along with anyone whose health condition was not under the management of a health care provider. Previous experience with telehealth was not necessary.

Participants were recruited through community health organizations. Patients receiving care from home care agencies in Missouri, being under the direct supervision of health care providers, were asked to volunteer for the study. Participants were categorized into 2 groups: adopters (those who had adopted telehealth for self-management) and nonadopters (those who had not adopted telehealth for self-management).

### Ethical Considerations

This study was reviewed and approved by the Capitol Technology University Institutional Review Board (approval IRB05242023a, approved on June 8, 2023). All participants provided informed consent prior to participation. Privacy and confidentiality were protected by institutional review board approval; data were deidentified and securely stored. No compensation was provided.

### Data Collection

A pilot study was conducted to establish the reliability of the semistructured interview, with 10 participants selected from the study population. These participants were then excluded from the main study. Data collected during the pilot study were scrutinized to determine the instrument’s capacity to collect data relevant and applicable to the study aims. The findings of the pilot study were used to review the data collection instrument and processes and implement any necessary instrument modifications to enhance its reliability.

Data were collected via semistructured interviews [17] with an open-ended question format. The following are sample questions:

(AU) Have you used telehealth services for diabetes management before? If yes, could you describe your experience with using telehealth? If not, can you explain the reasons for your decision not to use it?
(PE) How do you think telehealth can be useful for managing type 2 diabetes? Please explain.
(PE) What are the benefits of using telehealth for diabetes management?

Using a flexible interview schedule, participants engaged in these interviews through phone and video calls, which were digitally audio-recorded. Probes, follow-up questions, and comments were used to encourage participants to clarify statements where further information was required for a comprehensive understanding of experiences they described [17]. Through phone conversations and video calls, participants shared their experiences, challenges, and perceptions regarding the use of telehealth, especially those associated with management of T2DM.

### Data Demographics

Data gathered during the semistructured interviews included participants’ demographic information: race, sex, household size, household income, occupation, education level, and age. Data were collected on performance expectancy, indicating perspectives on their expectations of how telehealth might be helpful for the management of T2DM and the realization of health goals. Furthermore, data on effort expectancy were gathered, describing the participants’ perceptions of the ease of use of telehealth and factors they believed would affect the ease of use negatively and positively. Finally, data on the social and cultural influences on the use of telehealth for managing T2DM were collected, examining participants’ social networks (eg, family members, friends, and health care providers) and cultural factors (eg, preference for in-person care and language barriers).

Data regarding participants’ personal innovativeness, trust, behavioral intention, and actual use of telehealth services were also gathered. Personal innovativeness pertains to their comfort levels in using new technologies such as telehealth, while trust refers to their level of confidence in the security and privacy offered by telehealth applications and whether they had reservations about sharing their health information online. Behavioral intentions examined whether the participants would adopt telehealth for the management of T2DM and the factors that may influence those decisions. Finally, participants’ actual use of telehealth in the past was explored, with data collected on their associated experiences. For participants who did not have previous experience using telehealth, data were collected on their willingness to use it in the future.

### Data Analysis

Thematic analysis was employed to identify recurring patterns in participant responses. Qualitative data were categorized based on patterns that form specific themes; data excerpts were thereby organized within the concepts outlined in the UTAUT theoretical framework [18-20]. We first conducted line-by-line inductive coding of all transcripts to identify emergent concepts. These codes were grouped deductively under UTAUT constructs and refined through axial coding into subthemes. Finally, overlapping subthemes were consolidated into 4 primary barrier categories. Qualitative data from the interviews were examined using axial coding, enabling codes, subcategories, and categories contained in the participants’ perspectives to be more easily identified [19,21].

## Results

Thematic refinement yielded 4 consolidated barriers to telehealth adoption (language and cultural barriers, digital literacy and access, limited provider recommendations, and technology/infrastructure disparities), derived from 9 initial themes.

### Data Demographics

A total of 30 participants were recruited for the study: 15 adopters and 15 nonadopters to telehealth services. The 15 adopters were between the ages of 31 and 57, with the average age being 35 years. All of the adopters had attended institutions of higher education, with 6 having bachelor’s degrees, 4 having master’s degrees, and 2 having doctorates. All of the adopters were Asian American individuals, with multiple ethnicities represented, including Chinese, Indian, Vietnamese, Filipino, Korean, and Nepali. Men were disproportionately represented, with 12 men compared to only 3 women. The highest household income was US $200,000, while the lowest was US $36,000, with most households consisting of 3 people. Participants who were telehealth adopters also worked in various professions, including information technology, academia, and engineering, as well as in miscellaneous jobs (Table 1).

**Table 1.** Demographics of the adopters.

Adopter No.	Household size	Age (y)	Ethnicity	Gender	Household income (US $)	Occupation	Education level
1	4	45	Nepali	Male	200,000	IT professional	Master’s
2	4	39	Chinese	Male	90,000	Auto mechanic	Bachelor’s
3	5	38	Chinese	Male	150,000	Researcher	Bachelor’s
4	3	35	Indian	Male	80,000	IT professional	Bachelor’s
5	4	51	Korean	Female	100,000	Computer science	Bachelor’s
6	3	57	Korean	Male	150,000	IT networking	College
7	4	44	Vietnamese	Male	160,000	Professor	PhD
8	1	36	Filipino	Male	36,000	Sushi cook	College
9	4	46	Vietnamese	Female	120,000	Hair stylist	College
10	3	44	Chinese	Female	110,000	Academia	PhD
11	3	31	Vietnamese	Male	50,000	IT	Bachelor’s
12	2	35	Filipino	Male	75,000	Retired army personnel	Bachelor’s
13	4	49	Nepali	Male	175,000	Aircraft engineer	Master’s
14	3	45	Indian	Male	150,000	Civil engineer	Master’s
15	3	35	Indian	Male	108,000	Data engineer	Master’s

The 15 nonadopters of telehealth services were between the ages of 32 and 80, with the average age being 55 years. Participants in this group were less educated in comparison to the adopter group, with only 1 having a graduate degree. Five nonadopters graduated high school, while 9 had no formal education. However, as with the adopter group, there were various Asian American ethnicities represented in the group of nonadopters, including Nepali, Indian, Chinese, Filipino, Vietnamese, and Korean. There was less gender disparity among nonadopters compared to adopters, with 9 females and 6 males. The nonadopters earned much less than the adopters in terms of household income, which was between US $9000 and US $36,000. Most households had 2 people. Finally, 10 of the nonadopters were unemployed, while only 3 worked part-time (see Table 2).

**Table 2.** Demographics of nonadopters.

Nonadopter No.	Household size	Age (y)	Ethnicity	Gender	Household income (US $)	Occupation	Education level
1	1	80	Korean	Female	9000	Unemployed	High school
2	2	45	Korean	Female	12,000	Casual labor	High school
3	2	75	Chinese	Female	9000	Casual labor	High school
4	1	67	Indian	Male	9000	Unemployed	High school
5	2	62	Filipino	Female	11,400	Unemployed	No education
6	3	55	Nepali	Female	10,000	Unemployed	No education
7	3	67	Nepali	Male	11,000	Unemployed	No education
8	2	53	Indian	Female	12,000	Unemployed	No education
9	2	61	Vietnamese	Male	10,000	Unemployed	No education
10	2	38	Nepali	Male	15,000	Casual labor	No education
11	2	55	Vietnamese	Male	11,000	Unemployed	No education
12	3	32	Chinese	Female	36,000	Student	Graduate
13	2	36	Filipino	Male	36,000	Cook	High school
14	2	58	Indian	Female	12,000	Unemployed	No education
15	2	52	Nepali	Female	12,000	Unemployed	No education

Though past researchers have found facilitators to incorporating telehealth, this study only yielded barriers. To review, facilitators of telehealth implementation were videoconferencing, caregiver engagement, and delivery via the favored language of patients and caregivers [22]. Approaches to enhance telehealth consultations included in-person meetings to establish a relationship before shifting to telehealth and using text and audio telemonitoring to ensure that patients understood advice and instructions [22].

The interpretation following thematic analysis enabled the identification of 4 barriers to telehealth adoption among Asian American individuals with T2DM: (1) language and cultural barriers; (2) digital literacy and access; (3) limited provider recommendations; and (4) technology and infrastructure disparities. Beyond barriers, participants highlighted facilitators that can inform targeted implementation strategies, including provider recommendations, interpreter support, device access, insurance coverage, and perceived convenience.

### Language and Cultural Barriers

Nonadopters reported several problems and challenges with telehealth systems, with the most prevalent problem being a language barrier. Due to the lower level of education among nonadopters and most being first-generation immigrants to the United States, many could not speak English. The language barrier was an obstacle unless they found a doctor with whom they shared a common language. In fact, 10 of the 15 nonadopters spoke of the language barrier as the main challenge affecting their use of telehealth.

### Digital Literacy and Access

Effort expectancy involves the convenience and usability levels that adopters of telehealth experience when using the system [23,24]. Participants’ perceptions of the ease or difficulty of use of telehealth and any associated problems were explored, and a learning curve associated with initial use of telehealth services was identified.

Several adopters noted that there was a steep learning curve and they initially experienced significant difficulties. However, they quickly added that after using telehealth several times and/or having a doctor or a proficient family member explain its use, it eventually became easier to navigate. Some adopters, such as information technology professionals, did not have any problems using the telehealth system. Nonetheless, adopters mentioned some problems with the use of telehealth, such as not having access to the internet. Some telehealth systems are more complex than a simple call to the doctor and require the use of mobile apps accessible only through a smartphone connected to the internet. This issue affects accessibility, especially if the telehealth appointment is scheduled for a time when internet access is limited or not possible due to travel or other factors. Another problem associated with the use of telehealth relates to the availability of supporting technology, such as smartphones or other devices capable of complex operations (eg, camera-enabled desktop computers or laptops). Lack of these technologies represents a significant barrier to using telehealth.

### Limited Provider Recommendations

Though provider recommendations were limited, some participants did receive recommendations. Some adopters were forced by circumstances to use telehealth to aid in managing their diabetes, as was the case for P15:

*I was able to consult with a doctor over the phone to discuss my diabetes. I think useful; you can actually ask questions about your diabetes issues you have at any time.*

Some of those circumstances included constraints on health care access due to the COVID-19 pandemic, like P5, who said:

*My doctor also asked me to use telehealth. My doctor always communicates with me via text about my health issues. I think my doctor’s advice influenced me quite a bit. Because a doctor is a medical doctor, she knows what she’s doing. And she was strongly recommended, especially during COVID-19 time.*

Although they were required to use telehealth, the positive experience of doing so encouraged them to continue after the pandemic.

### Technology and Infrastructure Disparities

None of the nonadopters had ever used telehealth to assist them in their management of T2DM. There were various reasons given for nonuse, ranging from a lack of awareness of the existence of telehealth systems to not knowing how to use the system, like P2:

*I think training about telehealth and how to use it.*

However, some nonadopters also mentioned facing language barriers, which prevented them from using telehealth, like P7:

*No one said anything because of my language barrier.*

### Performance Expectancy

Performance expectancy is the degree to which a user believes that using telehealth will help them make gains in managing their health, for this study, T2DM specifically. Performance expectancy was assessed through examining participants’ thoughts on how using telehealth could help with their T2DM, as well as the perceived benefits of using telehealth.

Many adopters thought telehealth would introduce convenience and flexibility into the management of their T2DM because it eliminated travel to the doctor’s office. In addition, cost-effectiveness and time saved were mentioned by adopters as expected benefits of using telehealth. For example, adopters felt its use was cost-effective, saving them a trip to the doctor—and the associated expenses—while its capacity to provide secure and rapid access to health services ultimately saved valuable time. Adopters reported that using telehealth enhanced their access to health care services.

For nonadopters, performance expectancy was significantly lower compared to adopters. Specifically, there was a significant number of nonadopters who were not aware that telehealth might enhance diabetes management, perhaps because some were not familiar with telehealth systems. Nonetheless, many believed that using telehealth would be beneficial in helping them to manage their T2DM more effectively. Furthermore, when asked about the benefits of using telehealth, several nonadopters highlighted the convenience of not having to travel to the doctor’s office, reduced cost, and time efficiency as likely benefits. These benefits were similar to those mentioned by adopters. However, some nonadopters did not perceive that there would be any benefits associated with the use of telehealth, and their overall preference for in-person care was considerable. See Table 3 for adopters’ views on performance expectancy.

**Table 3.** Selected quotes by adopters about performance expectancy.

Participant	Adopter/nonadopter	Selected quotes
1	Adopter	But you still have the benefit of using telehealth. For example, it’s flexible because you do not have to travel to your doctor’s office, saving time.
3	Adopter	It is beneficial because I can call my doctor from anywhere. It is a benefit because I cannot travel and receive timely care for my problems.
5	Adopter	It was pretty seamlessly easy, I thought. Because I asked for the appointment, they asked me to fill out the information online, which I did pretty quickly. And then, about an hour later, they said they would call me back with the doctor. And so I got connected with the doctor, and I was able to consult with a doctor over the phone to discuss my diabetes. I think useful; you can actually ask questions about your diabetes issues you have at any time. Even though you’re not in the town, because sometimes I’m not in the United States. I am in Korea, visiting my parents or my friends in Korea I’m there for maybe three months or four months. I can just call or email my doctor. I can always connect with my doctor online or on the phone about my diabetes, and I don’t have any problem with that.
2	Adopter	Like I said, it’s convenient because I can call my doctor anywhere from a distance. It helps with remote monitoring and consultation of blood sugar levels. It is cost-saving because the gas price is going too high.
15	Adopter	I am very busy, and I do not have to drive to the doctor’s office. So it saves money and time. It’s a convenience.
13	Adopter	Like I said, using telehealth is helping me access the doctor faster. For example, it takes more than two months to get an appointment for the office visit, but it takes less than one week to make a telehealth appointment.
8	Adopter	Besides my diabetes problems and other health problems, telehealth increases access to my care in terms of fast service.
11	Adopter	It does help me to monitor my blood sugar. Using telemedicine increases my chances of improving my diabetes. I don’t have to always wait for your appointment.
12	Adopter	Honestly, it has been a lot more accessible, especially during the pandemic time. I can get a telehealth appointment within a week or so, but office visits take more than one month for an appointment.

### Data Analysis

The following 9 themes were identified using thematic analysis: actual use, performance expectancy, effort expectancy, social influence, facilitating conditions, cultural influence, personal innovativeness, trust, and behavioral intention.

#### Actual Use

Information was collected on whether participants had used telehealth to talk to their physicians about diabetes, their experiences, and, among those who reported not having used telehealth, the reasons for not using it. Findings showed that there was a high usage of telehealth among adopters, who felt that telehealth was beneficial. For example, P13 said:

*Like I said, using telehealth is helping me access the doctor faster. For example, it takes more than two months to get an appointment for the office visit, but it takes less than one week to make a telehealth appointment.*

Similarly, P15 said:

*I am very busy, and I do not have to drive to the doctor’s office. So it saves money and time. It’s a convenience.*

This group reported positive experiences that centered on the benefits of telehealth, including its convenience. Table 4 shows adopters’ views on actual use.

See Table 5 for a summary of nonadopters’ views on actual use.

**Table 4.** Selected quotes by adopters about actual use.

Participant	Adopter/nonadopter	Selected quotes
1	Adopter	I’ve used telehealth before for my diabetes management. I feel that telehealth is beneficial. I think it’s more convenient, easy to use, and as you start using it more and more.
4	Adopter	I have talked to my doctor by phone about my diabetes care. One thing I like about that is I don’t have to wait a month for the doctor. I can call them whenever I need to and discuss it with the doctor. It’s convenient that I do not have to wait too long.
11	Adopter	I have talked to my doctor before. Usually, either be on the phone or a Zoom call. It was easy for me after COVID-19; everything was on lockdown. So there is no choice.
10	Adopter	I have been using telehealth since the COVID-19 pandemic. Because I still feel the risk of exposure to the virus

**Table 5.** Selected quotes by nonadopters about actual use.

Participant	Adopter/nonadopter	Selected quotes
1	Nonadopter	I don’t know much about it. I never talked to my doctor using the phone or anything because I did not know there was a telehealth service available. I have no idea. My doctor did not say anything to me.
6	Nonadopter	I do not use it because of language barrier.
5	Nonadopter	I do not know how to use it. I never knew about it.

#### Social Influence

Social influence is the degree to which actors in a person’s social circles, such as the primary physician, family members, and friends, influence the decision of an individual to use telehealth. Participants were asked whether their doctors, friends, or family members encouraged the use of telehealth; they were then asked to describe the nature of that input.

Among adopters, the social influence of doctors and family members impacted their decision to start using telehealth. As doctors and family members communicated the benefits of using telehealth services, participants were led to consider it. When doctors described the benefits of convenience, cost-effectiveness, and time efficiency, they were influential in the participants’ decision. In addition to doctors, adopters indicated that family members also had an important role in encouraging them to adopt telehealth. Family members of participants indicated that there were tremendous benefits of using telehealth for managing diabetes and described it as an effective health care service that is gaining traction for treating this chronic illness.

In contrast, nonadopters did not receive advice from their doctors, friends, or family members regarding the use of telehealth. Some of the nonadopters believed that their nonproficiency in English was part of the reason their doctors never recommended telehealth. Others stated that their physicians did not suggest it.

#### Facilitating Conditions

Facilitating conditions concerns the user’s belief that the technical infrastructure and other conditions necessary for the use of telehealth already exist [25]. Participants’ perceptions regarding whether the necessary resources to facilitate telehealth use for diabetes care were in place as well as their perceptions regarding whether they had access to necessary devices were examined.

Regarding owning the necessary devices, all adopters reported that they owned a smartphone, allowing them to easily connect to the internet and run the mobile apps necessary to use telehealth effectively. In addition, insurance coverage was mentioned by adopters as a necessary support enabling the use of telehealth. Several adopters reported that their insurance did not cover the service and that they had to pay out-of-pocket. This finding highlighted the need for insurance coverage as one of the facilitating conditions to enable adopters to use telehealth services when needed for managing diabetes. Adopters also reported that they would like support in the form of training on effective use of telehealth in general, as well as for diabetes management, specifically. There was a perception that doctors and their staff could also benefit from such training.

Nonadopters faced conditions that did not facilitate their use of telehealth. For instance, a majority of nonadopters did not own the devices, such as smartphones or laptops, required for telehealth access. In addition, other nonadopters deemed the services of a translator to be an important support resource necessary for them to use telehealth services effectively. This finding was logical, considering that 10 of the 15 nonadopters identified language as a barrier limiting their use of telehealth. Furthermore, most nonadopters pointed out that financial health was an important aspect for them to consider in relation to telehealth services. Some reported social security as their sole source of income. Those who indicated that financial assistance was necessary to support their use of telehealth services reported they would use such aid to purchase a phone and pay for the services of an interpreter. In addition to financial aid, they reported a need for training on telehealth and how it applies to diabetes management. See Table 6 for a summary of nonadopters’ views on facilitating conditions.

**Table 6.** Selected quotes by nonadopters about facilitating conditions.

Participant	Adopter/nonadopter	Selected quotes
5	Nonadopter	I have no smartphone or computer.
9	Nonadopter	I have no phone or computer.
7	Nonadopter	Need an interpreter like you and pay for an interpreter’s services.
11	Nonadopter	Need help with an interpreter.
4	Nonadopter	I survive from my social security check.
8	Nonadopter	Financial help to by phone and an interpreter.
3	Nonadopter	Education about telehealth and training about how to use it.
2	Nonadopter	I think training about telehealth and how to use it.
13	Nonadopter	Training about telehealth in the community or clinic.

#### Cultural Influence

Although the study participants were from the Asian American community in general, there were multiple ethnicities represented. One cultural influence identified by adopters was a preference for in-person care. However, even though adopters preferred in-person care, a recommendation by a doctor to use telehealth overrode the cultural-based preference of some adopters. It was clear that the convenience of using telehealth had more influence on their decision regarding whether to adopt telehealth. In contrast, other adopters retained their preference for in-person care despite the benefits of telehealth. Language was not an issue for most adopters, especially among second-generation Asian American individuals. Likewise, language was not an issue for Asian American individuals who spoke the same non-English language as their doctor. Still, some adopters did experience language barriers that affected their effective use of telehealth services.

Most nonadopters were not proficient in English, and this adversely affected their ability to use telehealth systems. Therefore, among participants who chose not to use telehealth, the primary concern was the language barrier; several nonadopters specifically cited the language barrier as prohibitive. A preference for in-person care was also cited as part of the reason participants did not adopt telehealth services.

#### Personal Innovativeness

Personal innovativeness is related to participants’ comfort in using new technologies and their willingness to learn how to use telehealth [26,27]. All adopters were comfortable with telehealth technology. Participant occupations were often related to comfort level with telehealth systems. As many adopters were professionals in fields where the use of different technologies was required, they were easily able to attain proficiency on different platforms. Other adopters decided they were comfortable with new technologies because they were already using telehealth.

Among adopters, there was an overwhelming willingness to learn how to use telehealth for diabetes care. As all adopters except one were already using telehealth, they were willing to commit themselves to learning about the functionality and use of the system in even more detail.

In contrast, many nonadopters were not comfortable with new technologies due to legacy challenges, such as the language barrier. Some nonadopters who could not read or write in English would undoubtedly experience challenges using telehealth. However, regardless of language concerns, nonadopters were willing to learn how to use the technology.

#### Trust

Adopters must trust in the security, privacy, and confidentiality of their health information stored in health care systems to continue using telehealth services. Most adopters reported that they were comfortable with the security and privacy of their health information, expressing their confidence as being attributable to Health Insurance Portability and Accountability Act policies. Nonetheless, some adopters had concerns regarding security, as they reported worrying about the safety of their identifying information. In addition, some adopters were concerned that others would see their sensitive health information, although there were a few who felt that they could trust their physicians to keep their information safe and protected.

Most nonadopters did not trust telehealth systems. Their concerns were related to sharing their health information online and the security and privacy of information disclosed while using the systems. However, several nonadopters did not have an opinion one way or the other, as they had never used telehealth systems.

#### Behavioral Intention

Behavioral intention relates to the decision to continue or discontinue use of telehealth and the reasons for this decision [28]. All adopters except one said they would continue to use telehealth systems. The reasons that the majority of participants decided to continue using telehealth services were attributed to the benefits it provided. The one adopter who was noncommittal mentioned safety and security concerns about health information stored in the telehealth systems. Despite such concerns, most adopters were willing to continue using telehealth as a modality for managing diabetes.

Even though nonadopters had never used telehealth systems and despite the significant challenges identified, they were positive in their intentions toward the use of telehealth. A majority of nonadopters were willing to learn more about telehealth if someone taught them. In fact, nonadopters attributed their behavioral intentions to the need to improve their diabetes care. Other nonadopters cited the benefits of telehealth such as convenience or saving time and money as the reasons for their desire to learn. However, a few nonadopters refused to use telehealth in the future, citing existing language barriers.

## Discussion

### Summary of Key Findings

The analysis of the emergent themes from the qualitative data yielded 9 themes. The themes were actual use, effort expectancy, performance expectancy, social influence, facilitating conditions, cultural influence, personal innovativeness, trust, and behavioral intentions. Several subthemes also emerged from the themes. They included the possession of access devices, language barrier, the convenience and ease of using telehealth systems, cost and time effectiveness resulting from using telehealth systems, the role of physicians in recommending the use of telehealth systems, the need for training and education on the use of telehealth systems, insurance coverage of telehealth services, and an intention to continue using or start using telehealth systems.

Regarding actual use, the findings showed that the adopters who used telehealth systems cited the convenience, cost and time efficiency, and flexibility as some of their reasons for using telehealth systems, corresponding to Hu et al’s [29] findings, showing that access to technology was a notable determinant in patients’ interest in mobile health interventions. The nonadopters attributed their lack of awareness of the existence of telehealth systems and how to use them as some of the reasons for not using telehealth systems for the management of T2DM. Regarding effort expectancy, the findings showed that the participants perceived the use of telehealth systems to be easy. Concerning performance expectancy, telehealth systems improved convenience, enhanced access to health care services, were cost-efficient, saved time, and enabled flexibility in access to services. Regarding social influence, the findings showed that physicians, friends, and family played a significant role in promoting the adoption of telehealth. Similarly, Mora and Golden [30] found that family strongly influences individuals’ diabetes management plans. Recommendations of telehealth from physicians, family, and friends were conspicuously absent among the nonadopters, while the adopters reported the influence of those recommendations on their decisions to adopt telehealth systems.

The facilitating conditions identified as being influential to the adoption of telehealth systems for the management of T2DM included access to devices, such as smartphones and computers. The coverage of telehealth services by insurance providers was another important facilitating condition. The other conditions were training on how to use telehealth systems and education on the use of telehealth systems for the management of T2DM. The findings also showed that cultural factors influenced the adoption of telehealth systems. The two most influential cultural factors were the language barrier and a preference for in-person care. Mora and Golden [30] also found that language barriers impacted diabetes management approaches.

Findings on personal innovativeness showed that there was an equal share of comfort and discomfort in using telehealth systems. The findings also showed that legacy challenges, such as the language barrier and lack of access to devices, affected the participants’ ability to use telehealth systems. Regarding trust, participants shared concerns regarding the privacy, security, safety, and confidentiality of the health information stored in telehealth systems. However, there was also trust in the professionalism of physicians and the safeguards provided by the Health Insurance Portability and Accountability Act policy. Concerning behavioral intentions, there was an overwhelming desire to continue using telehealth systems. Even those who had not used telehealth systems before were willing to use them, provided they were trained and offered financial aid through which to acquire access devices. The behavioral intentions of the participants were informed by the benefits of convenience, time savings, cost efficiency, and enhanced access to health care services.

The findings showed that cultural influences played a role in the participants’ decision to adopt or not adopt telehealth systems for the management of T2DM. The most significant cultural factor influencing the decision not to use telehealth among the nonadopters was the language barrier. Research has shown that most of the telehealth platforms used in the United States use the English language [31]. The implications are that populations that are not fluent in English might find telehealth systems to be of limited benefit. This was certainly the case for many nonadopters. To address this challenge, the researcher recommends culturally-adapted telehealth systems that target underserved racial and ethnic minorities. Culturally adapted telehealth systems will not only address the language issue but also incorporate other cultural adaptations that would enhance the usability of telehealth systems for racial and ethnic minorities. Existing research supports this recommendation [32].

### Barriers to Telehealth Adoption

The results of this qualitative case study indicate that language and cultural barriers significantly impact Asian American individuals’ use of telehealth for managing T2DM. Language and cultural barriers have been recognized as key obstacles in health care access, often limiting patient engagement. For example, there is a limited availability of translated telehealth materials, and the scarcity of bilingual health care providers and interpreters makes it even more difficult for many groups to use this technology. When considering the culture of Asian American people, it also seems that there is a preference for in-person consultations with health care providers.

Another barrier to using telehealth is digital literacy and access, as many Asian American people do not have access to either technical support or patient education resources. Older adults especially seem to struggle with navigating telehealth platforms. Without proper training and support, these individuals remain excluded from telehealth-driven diabetes management.

The findings also highlight the limited recommendations of telehealth services by providers, which could be better promoted as a way for patients to improve their management of T2DM. Health care provider recommendations play a crucial role in shaping patient perceptions of telehealth. As the study uncovered, health care providers often do not actively recommend telehealth services, so many patients may not be aware of this option. Consequently, patients are also unaware of the many benefits of using telehealth services, which represents a significant barrier to its adoption. This highlights the need for provider engagement strategies to integrate telehealth into routine diabetes management.

Finally, technology and infrastructure disparities exacerbate other barriers to the use of telehealth services. Low-income individuals struggle with the cost of high-speed internet and smart devices, widening the gap of health care inequity [9]. For example, many patients, especially those with low income, are not able to obtain the devices (eg, smartphones and laptops) needed to access telehealth. Add internet connectivity issues and it is no surprise that many patients do not use telehealth. Addressing these disparities requires policy intervention that expands broadband access and subsidizes telehealth technology for underserved communities. Overall, the findings from this study align with existing literature on telehealth disparities among minority populations [11].

### Limitations

Adopters were generally younger, more educated, and higher-income than nonadopters. These socioeconomic differences likely confound the observed adoption patterns. While our sample size precluded stratified or adjusted analyses, future studies should employ matched sampling or multivariable adjustment to disentangle cultural influences from socioeconomic status. Participants were recruited through two community-based health organizations in Missouri: A Federally Qualified Health Center and a local Asian-serving nonprofit clinic. Both provided limited interpreter support, which shaped recruitment feasibility and participant diversity.

The language barrier was a significant limitation during the collection of data. The participants were drawn from various ethnicities. Therefore, they had diverse native languages. Many were not proficient in English and did not share a common language with the researcher. The researcher relied on the services of an interpreter to translate the question to the participant and the response from the participant back to the researcher. The translation is prone to loss of meaning because the interpreter must interpret and decode the participant’s words to derive their meaning. Context, whether personal or cultural, is important to the meaning of the participants’ words. Furthermore, facial expressions, gestures, tone of voice, and pauses are also central to meaning. While the interpreter may translate the words said by the participant, an accurate translation of the meaning, semantics, and nuances behind the words may not always be possible. Therefore, some or the entire meaning of the communication may be lost during the translation.

The knowledge level of the nonadopters about telehealth systems is a limitation to the value of the data gathered from the cohort. Most of the participants were unaware of the existence of telehealth systems. Therefore, it is possible that they did not actually decide not to adopt telehealth for the management of their T2DM. The implications of this lack of awareness are that the information they provided may not have reflected the influence of the unique characteristics of telehealth on their nonadoption but rather an influence of their lack of awareness of the existence of telehealth systems and the value they provide. Lack of awareness of telehealth systems may explain responses to several prompts, most of which revolved around “I do not know.” The value of this information in answering the research questions was limited.

### Conclusions

This qualitative case study identified unique characteristics, supported by the UTAUT model, that influenced the adoption intent and adoption of telehealth among Asian American people with T2DM in Missouri. Overall, there were many valuable insights into the cultural and technological barriers facing Asian American people when using telehealth.

## Abbreviations

T2DM: type 2 diabetes mellitus
UTAUT: unified theory of acceptance and use of technology
